# Development of lung function in very low birth weight infants with or without bronchopulmonary dysplasia: Longitudinal assessment during the first 15 months of corrected age

**DOI:** 10.1186/1471-2431-12-37

**Published:** 2012-03-23

**Authors:** Gerd Schmalisch, Silke Wilitzki, Charles Christoph Roehr, Hans Proquitté, Christoph Bührer

**Affiliations:** 1 Department of Neonatology, Charité University Medicine, Berlin, Germany; 2 Department of Neonatology, Charité University Medicine, Charitéplatz 1, D-10117 Berlin, Germany

## Abstract

**Background:**

Very low birth weight (VLBW) infants (< 1,500 g) with bronchopulmonary dysplasia (BPD) develop lung damage caused by mechanical ventilation and maturational arrest. We compared functional lung development after discharge from hospital between VLBW infants with and without BPD.

**Methods:**

Comprehensive lung function assessment was performed at about 50, 70, and 100 weeks of postmenstrual age in 55 sedated VLBW infants (29 with former BPD [O_2 _supplementation was given at 36 weeks of gestational age] and 26 VLBW infants without BPD [controls]). Mean gestational age (26 vs. 29 weeks), birth weight (815 g vs. 1,125 g), and the proportion of infants requiring mechanical ventilation for ≥7 d (55% vs. 8%), differed significantly between BPD infants and controls.

**Results:**

Both body weight and length, determined over time, were persistently lower in former BPD infants compared to controls, but no significant between-group differences were noted in respiratory rate, respiratory or airway resistance, functional residual capacity as determined by body plethysmography (FRC_pleth_), maximal expiratory flow at the FRC (V'max _FRC_), or blood gas (pO_2_, pCO_2_) levels. Tidal volume, minute ventilation, respiratory compliance, and FRC determined by SF6 multiple breath washout (representing the lung volume in actual communication with the airways) were significantly lower in former BPD infants compared to controls. However, these differences became non-significant after normalization to body weight.

**Conclusions:**

Although somatic growth and the development of some lung functional parameters lag in former BPD infants, the lung function of such infants appears to develop in line with that of non-BPD infants when a body weight correction is applied. Longitudinal lung function testing of preterm infants after discharge from hospital may help to identify former BPD infants at risk of incomplete recovery of respiratory function; such infants are at risk of later respiratory problems.

## Background

Bronchopulmonary dysplasia (BPD) remains the most common long-term complication of very preterm birth [1], despite the widespread use of prenatal steroids, exogenous surfactants, and minimally invasive strategies of respiratory support [2-4], along with other advances in neonatal care [5-7]. In contrast to what was noted in the pre-surfactant era, when BPD was characterized by airway inflammation, fibrosis, and smooth muscle hypertrophy, the "new BPD" is associated with delayed alveolar and vascular development, resulting in simplification of alveolar structures, dysmorphic capillary configurations, and variable extents of interstitial cellularity and fibroproliferation [8,9].

A considerable body of data has revealed that very preterm infants with "new BPD" exhibit abnormalities in lung function after birth [10-12], during the first years of life [13-17], throughout childhood [7,18-23], and into early adolescence [24]. It is currently unclear whether survivors of BPD are at increased risk of developing a later COPD-like phenotype [25].

Most previous studies of lung function in preterm infants with BPD have been limited by variations in methods, equipment, and outcome measures. Further, the lack of controls or appropriate reference data have hampered the interpretation and comparability of results [26]. Therefore, data are often inconsistent because of methodological differences among studies. Moreover, most lung function studies performed during childhood have focused primarily on assessment of small airway performance. However, BPD also arrests alveolar and vascular development, such that abnormalities subsequently develop in the distal lung parenchyma [5]. Despite the extensive literature on lung function in children that had BPD in infancy, little is currently known about either pulmonary growth in such children or the ability of the very immature lung to recover from BPD. It seems essential to determine the extent of possible catch-up growth, and, most importantly, to identify parameters of lung function that indicate the presence of BPD-specific impairment. Therefore, the aim of the present longitudinal study was to compare the development of lung function and somatic growth in very preterm infants with and without BPD during the first 15 months of corrected age.

## Methods

### Subjects

For the present retrospective analysis, we identified 55 preterm infants of birth weight < 1,500 g who had undergone serial lung function testing (LFT) at three time points (at about 50, 70, and 100 weeks of postmenstrual age) in our outpatient lung function laboratory. Infants with congenital diaphragmatic hernia, congenital heart disease, neuromuscular disease, or thoracic wall deformities, were excluded from the study. Of the 55 infants born between October 1995 and February 2010, 29 had been diagnosed with BPD, based on a requirement for supplemental oxygen at 36 weeks of postmenstrual age. Written parental consent was obtained before LFT. The study was approved by our Institutional Data Safety Committee.

### Lung function testing (LFT)

Measurements were performed on clinically stable children who had not experienced any respiratory tract infection over the 3 weeks prior to testing. Before LFT, body weight was measured to the nearest 10 g (Seca, Hamburg, Germany); body length from crown to heel was measured to the nearest 5 mm using an inelastic tape; and, (at the end of LFT), an arterialized capillary blood gas sample was taken (ABL800 FLEX Radiometer; Brønshøj, Denmark).

After temperature stabilization for at least 30 min, all equipment used was calibrated prior to each measurement according to the recommendations of the manufacturer. When LFT was planned, sleep was induced by oral administration of chloral hydrate (50 mg^.^kg^-1^) 15-30 min before testing. Each sleeping infant was placed in the supine position with the neck in a neutral position, supported by a neck roll. After a pause of 5-20 min, tidal breathing parameters [tidal volume (V_T_); respiratory rate (RR); and minute ventilation (V'_E_) were measured using the dead-space free flow-through technique employing customized equipment that has been described in detail elsewhere [27]. Next, lung mechanical parameters (respiratory compliance [C_rs_] and respiratory resistance [R_rs_]) were measured using the occlusion test. Airway resistance (R_aw_) and functional residual capacity (FRC_pleth_) were assessed using a constant volume infant plethysmograph (Jaeger, Würzburg, Germany). Employing the same equipment, the maximal expiratory flow at the functional residual capacity (V'max_FRC_) was measured using the rapid thoraco-abdominal compression technique, in line with international guidelines [28].

Finally, multiple breath inert gas washout was performed using 5% (v/v) sulfur hexafluoride (SF_6_) (Ecomedics AG, Dürnten, Switzerland) as the tracer gas; this measured the proportion of the lung volume that participated in gaseous exchange (FRC_SF6_). During all pulmonary function tests, heart rate and oxygen saturation level were continuously monitored via pulse oximetry (N-200; Nellcor, Hayward, CA).

### Statistical methods

Patient characteristics are presented as proportions (% values) or as means with standard deviations (SDs; in brackets) and were compared using Fisher's exact test or either the paired or unpaired t-test, as appropriate. Parameters of lung function are shown as group means, with 95% confidence intervals (CIs), in both the text and the Figures. The effect of BPD on development of lung function parameters was explored by multivariate analysis of variance (MANOVA); gestational age and birth weight were used as covariates. Statistical analysis was performed using SPSS (version 19; SPSS Inc. Chicago, IL). A *p *value of < 0.05 was considered to be statistically significant.

## Results

### Characteristics of the study population

Table 1 compares the characteristics, at birth, of infants with and without BPD. The former BPD infants were of lower gestational age and birth weight; the proportion of such infants that had an extremely low birth weight (< 1,000 g) was almost 3-fold higher than in the control group. The proportions of infants treated with surfactant, and the frequency of prenatal steroid administration, did not differ significantly between the two groups. All BPD infants required invasive mechanical ventilation (MV) during the neonatal period whereas only half of non-BPD infants were mechanically ventilated.

**Table 1:** Patient characteristics in the neonatal period, shown as means with SDs (in brackets) or as numbers with percentages (%).

	Without BPD	With BPD	*p*-value
	N = 26	N = 29	
Gestational age (weeks)	29.08 (2.12)	26.41 (2.19)	**< 0.001**

Birth weight (g)	1124.1 (248.3)	815.7 (243.1)	**< 0.001**

Birth weight < 1,000 g	7 (27%)	25 (86%)	**< 0.001**

Fetal lung maturation^1)^	14/19 (74%)	12/17 (71%)	1.000

Surfactant administration^1)^	18/20 (90%)	18/20 (90%)	1.000

Mechanical ventilation	15 (58%)	29 (100%)	**< 0.001**

Mechanical ventilation for ≥ 7 d	2 (8%)	16 (55%)	**< 0.001**

Statistically significant *p*-values are shown in bold

1) Total number is reduced because the some data of outpatients were incomplete

### Somatic growth

**Figure 1:**
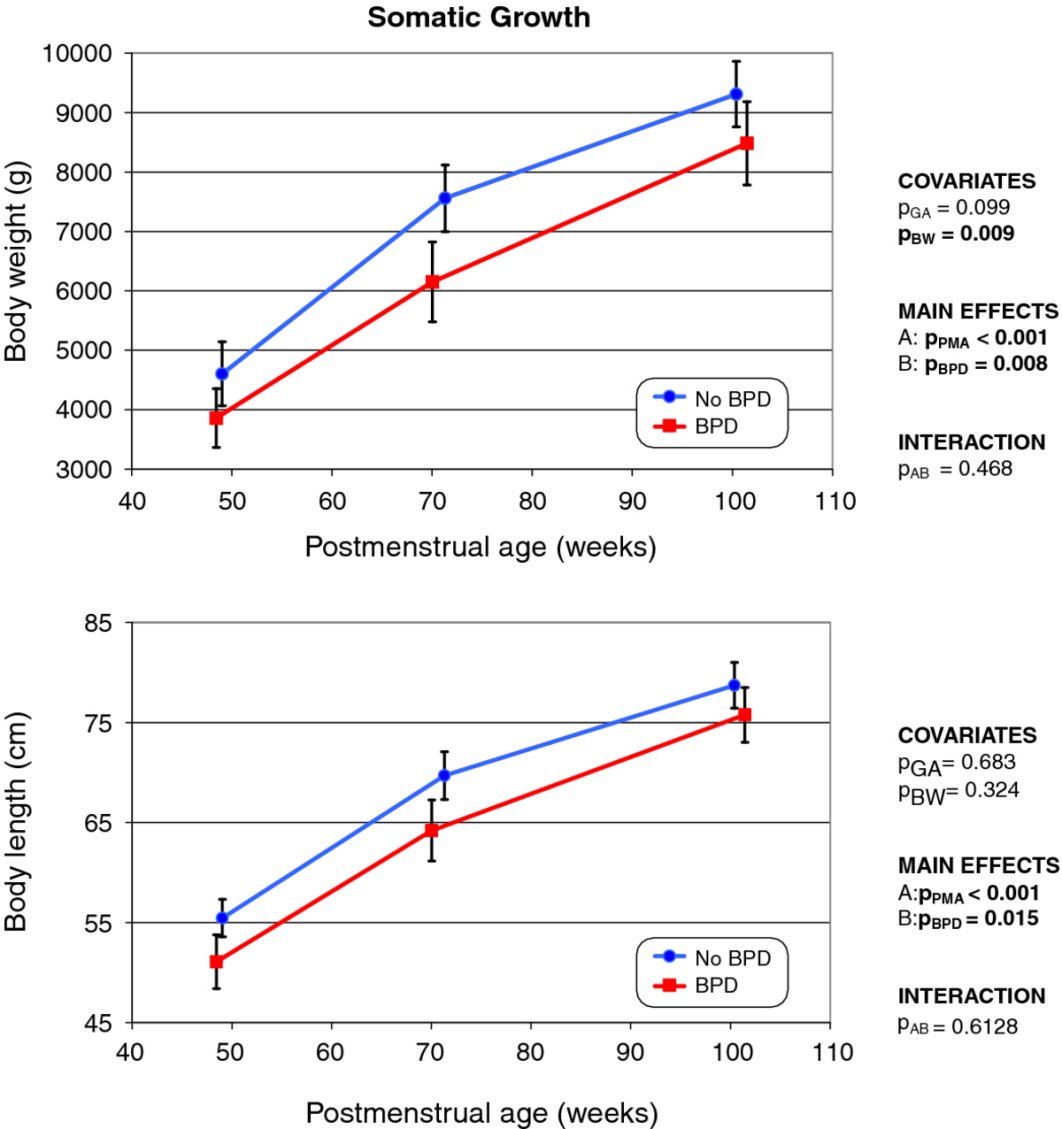
**Changes over time in body weight (top) and body length (bottom) of infants with and without former BPD (means with 95% CIs) The *p*-values show the extent of statistical significance of the covariates gestational age (p_GA_) and birth weight (p_BW_); those of the main effects by postmenstrual age (p_PMA_) and BPD (p_BPD_); and that of the interaction (p_AB_) of both factors**. Statistically significant values are shown in bold.

Age at the day of LFT is shown in Table 2. No statistically significant difference was evident between the two groups in either chronological or postmenstrual age. In contrast, body weight (*p *= 0.008) and body length (*p *= 0.015) were lower in BPD infants than in controls (Figure 1). MANOVA revealed that birth weight significantly influenced the rate of gain of body weight over time (*p *= 0.009). The observed differences in body weight and length between the two groups remained constant at all three test time points. No statistically significant interaction was evident between BPD and PMA.

**Table 2:** Chronological and postmenstrual age on the day of lung function testing (LFT) (means with SDs in brackets; the *p*-values show the extent of statistical significance when data from the two patient groups were compared)

	1^st ^LFT	2^nd ^LFT	3^nd ^LFT	*p*-value
*Age (days)*				

Without BPD	140.1 (48.5)	302.7 (77.7)	522.3 (128.6)	
	
With BPD	150.7 (49.1)	293.0 (115.2)	517.1 (155.5)	0.928

*Postmenstrual age (weeks)*				

Without BPD	49.0 (7.5)	71.3 (12.8)	100.4 (22.1)	
	
With BPD	48.4 (8.0)	70.0 (17.6)	101.4 (22.6)	0.944

### Tidal breathing

**Figure 2:**
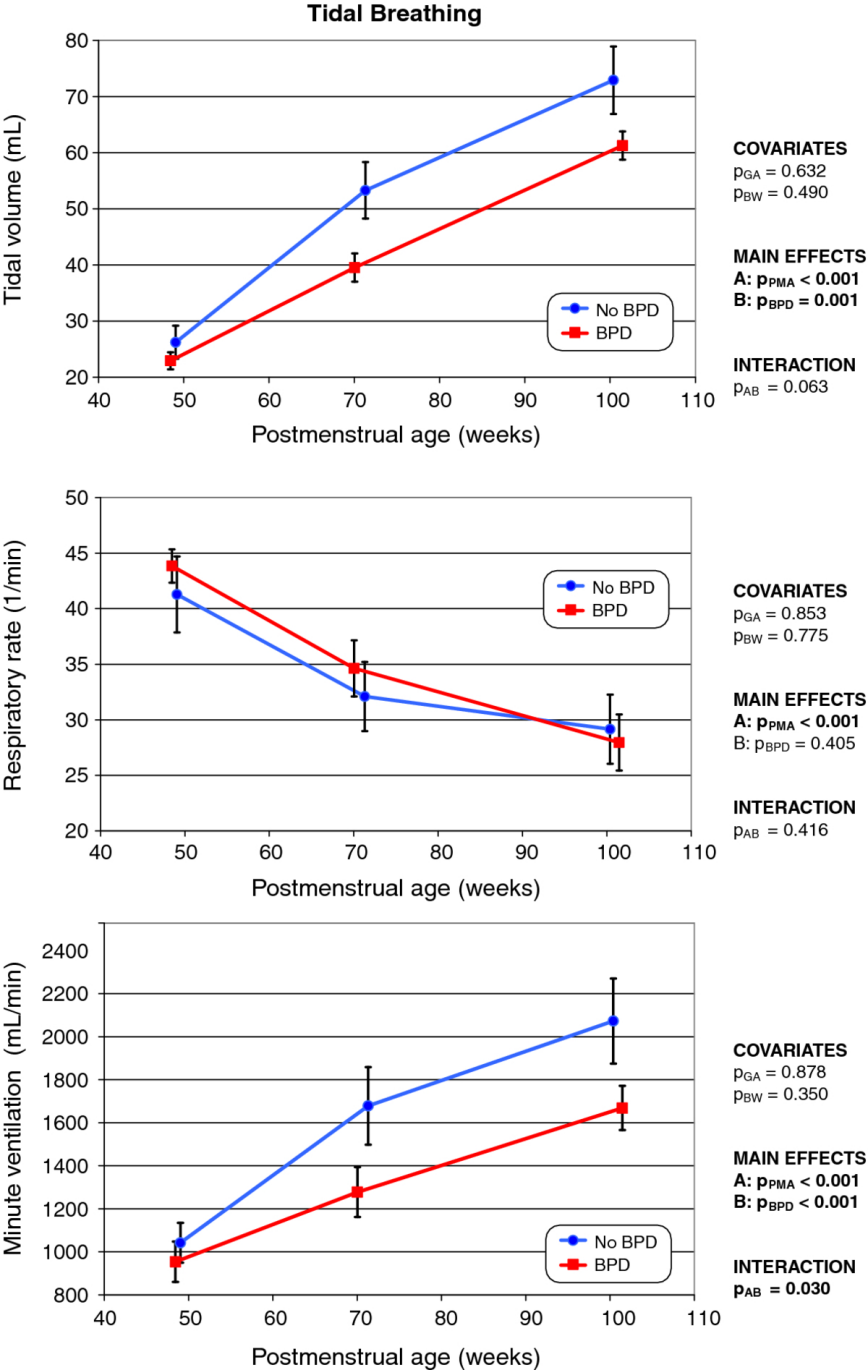
**Changes over time in tidal volume (top), respiratory rate (middle), and minute ventilation (bottom) in infants with and without former BPD (the mode of presentation is the same as that of Figure 1)**.

All tidal breathing parameters (Figure 2) showed rapid development (*p *< 0.001) in both groups. In BPD infants, both V_T _and V'_E _were significantly lower (*p *≤ 0.001) than in non-BPD infants, but no statistically significant difference in the respiratory rate was evident. Neither gestational age nor birth weight significantly influenced the values of the tidal breathing parameters. However, V'_E _showed significant interaction (*p *= 0.03) with BPD and PMA: V'_E _increased more rapidly in non-BPD infants compared to those with former BPD. This was particularly evident when data from the first and second LFT session were compared.

**Figure 3:**
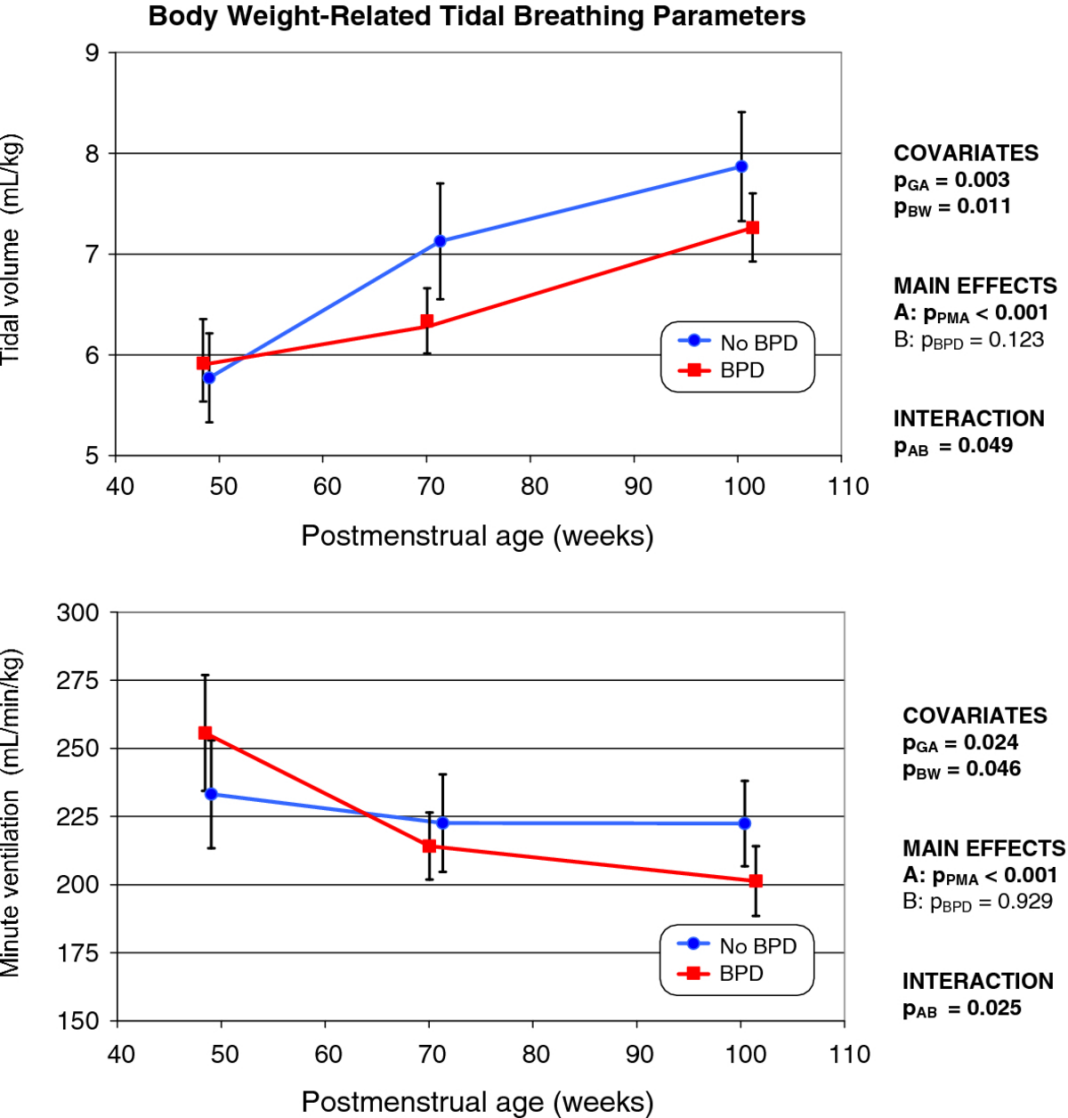
**Changes over time in tidal volume (top), minute ventilation (bottom), normalized to actual body weight, in infants with and without former BPD (the mode of presentation is the same as that of Figure 1)**.

After normalization of V_T _V'_E _to actual body weight (Figure 3), the between-group differences became statistically insignificant. However, gestational age and birth weight exerted statistically significant impacts on both weight-related parameters studied. Further, the interaction between BPD and PMA remained statistically significant upon normalization by body weight, indicating that the development, over time, of the weight-related parameters breathing parameters V_T _and V'_E _differed. As shown in Figure 3 V'_E _related to the body weight remained stable in non-BPD infants but decreased continuously in former BPD infants, because V_T _was lower in such infants.

### Lung mechanics

**Figure 4:**
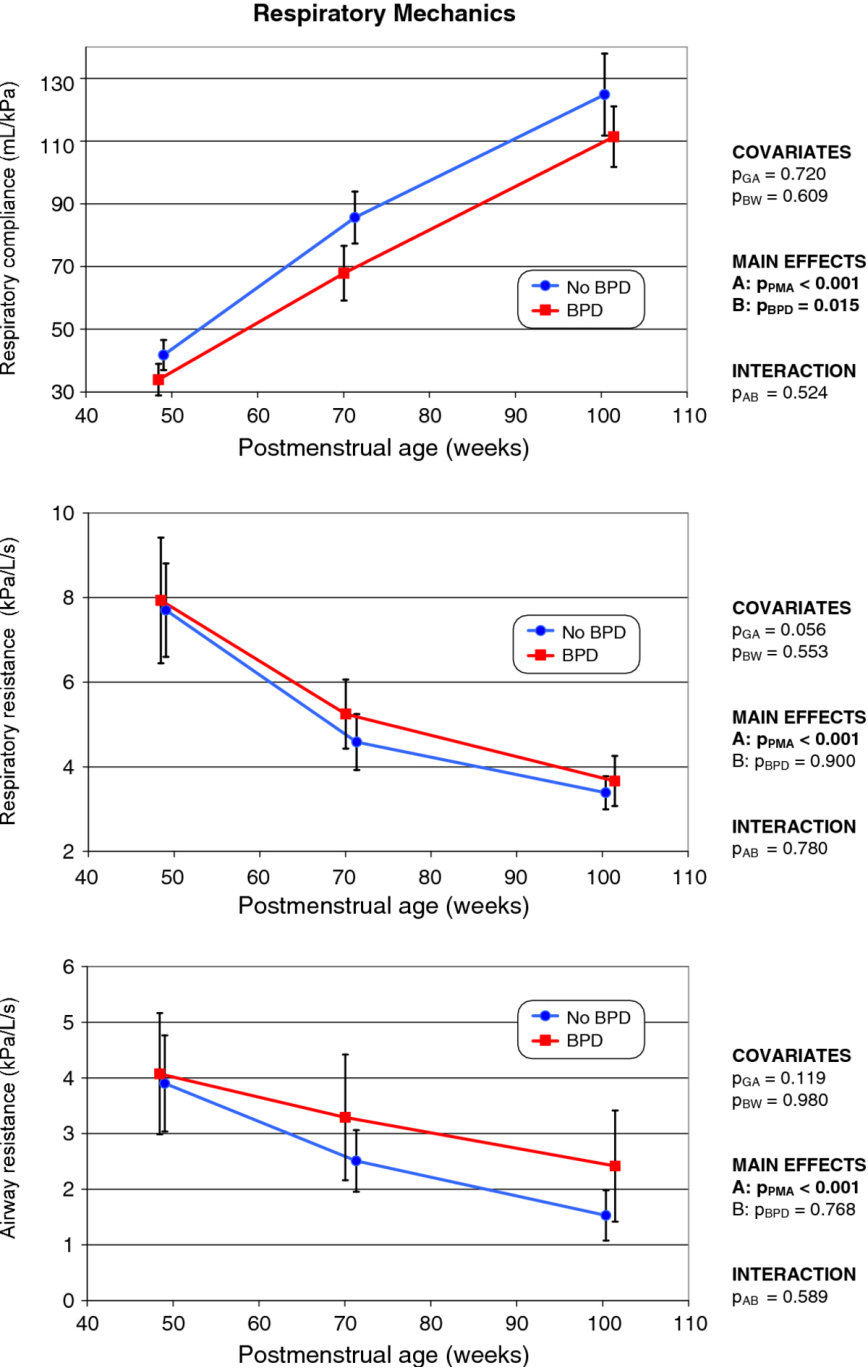
**Changes over time in respiratory compliance (top), respiratory resistance (middle), and airway resistance (bottom), in infants with and without former BPD (the mode of presentation is the same as that of Figure 1)**.

**Figure 5:**
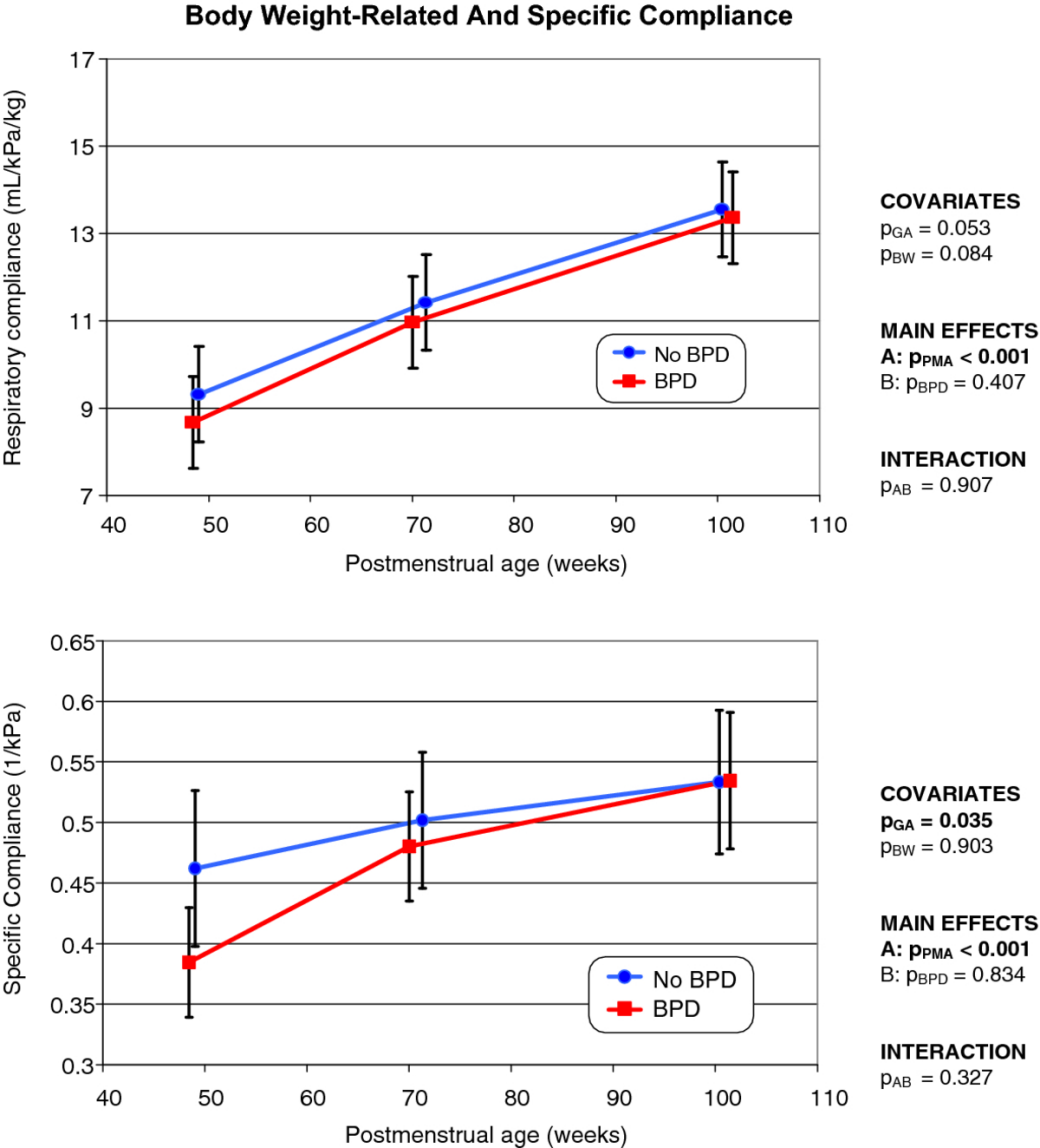
**Changes over time in respiratory compliance normalized to actual body weight (top), and specific compliance (bottom), in infants with and without former BPD (the mode of presentation is the same as that of Figure 1)**.

As with the tidal breathing parameters, the development over time of all lung mechanical parameters differed significantly (*p *< 0.001) between the two groups (Figure 4), but the covariates did not significantly influence such variations. In former BPD infants, C_rs _was significantly lower than in non-BPD infants, but rose in parallel as PMA increased. After normalization of C_rs _values to actual body weight (Figure 5), the differences between the groups became statistically insignificant; both groups developed similarly. Specific compliance (C_RS_/FRC; the elasticity of a unit of lung volume) increased in both groups, but apparently more rapidly in former BPD infants (Figure 5). At 15 months of age, the specific compliance was nearly identical in either group.

Both R_rs _and R_aw _(Figure 4) decreased with increasing age (*p *< 0.001). Although the mean values of R_rs _and R_aw _were always somewhat higher in former BPD infants compared to non-BPD infants, such differences never attained statistical significance.

### V'max_FRC_

**Figure 6:**
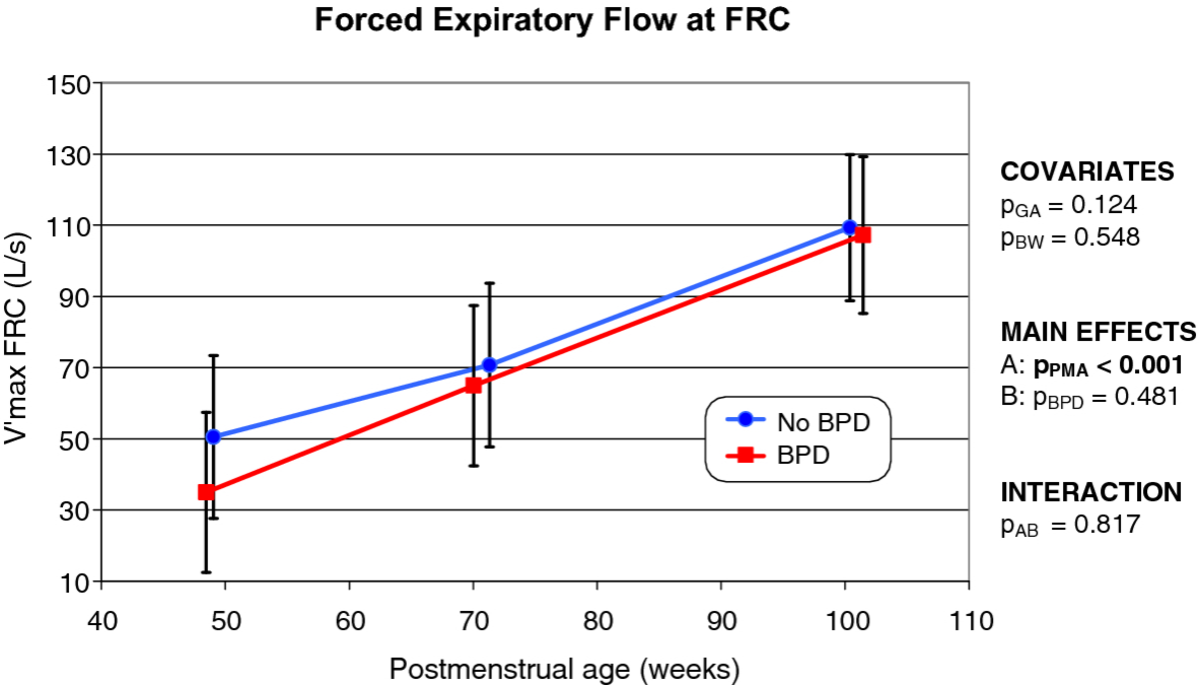
**Changes over time in the forced expiratory flow rate, at the FRC, in infants with and without former BPD (the mode of presentation is the same as that of Figure 1)**.

In both non-BPD and BPD infants, V'max_FRC _increased rapidly over time (*p *< 0.001); the value more than doubled between the first and third LFT sessions (Figure 6). At a PMA of approximately 100 weeks, the mean V'max_FRC _of either patient group was near-identical.

### Functional residual capacity

**Figure 7:**
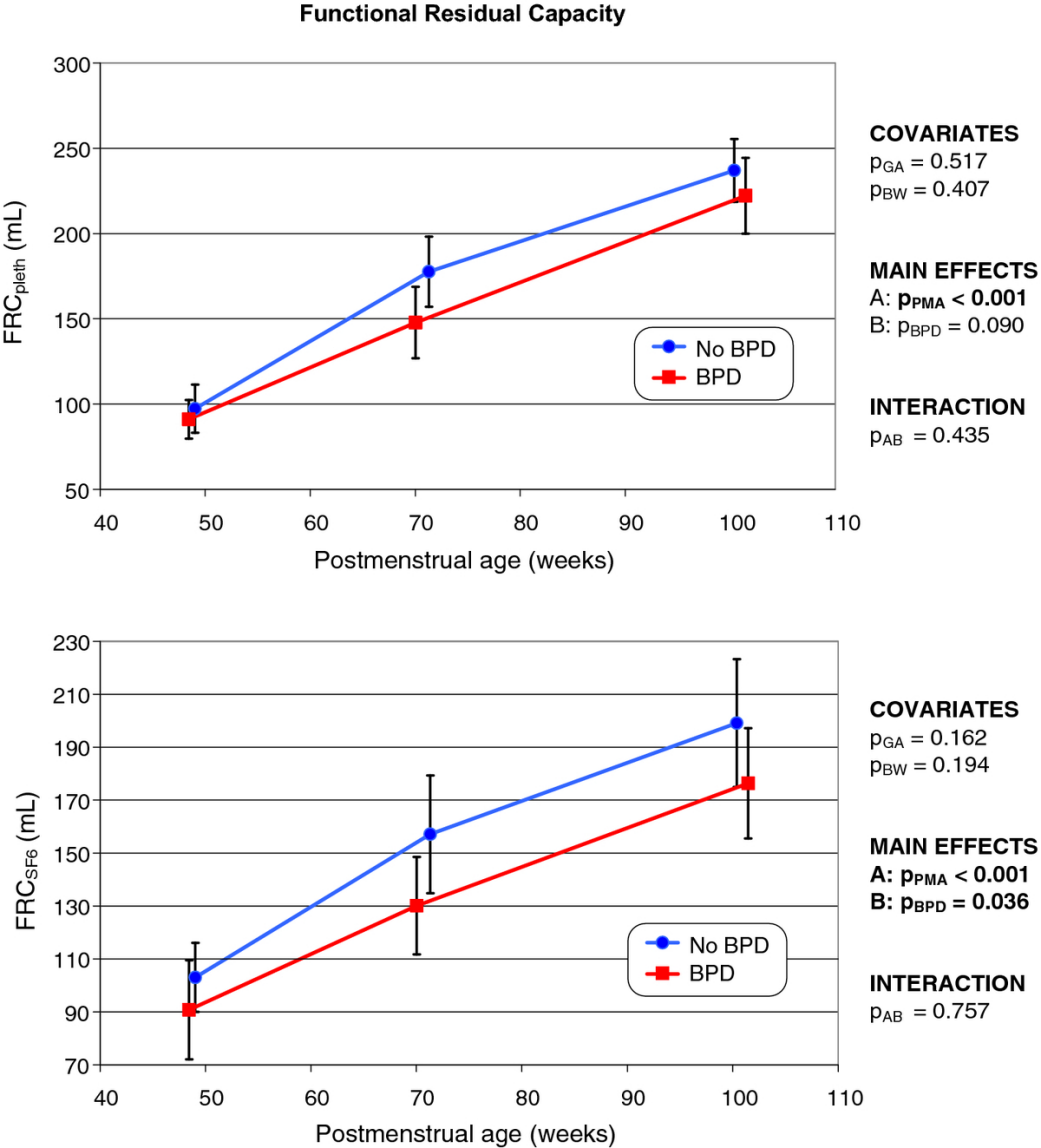
**Changes over time in functional residual capacity as measured by body plethysmography (top), and the SF_6 _multiple breath washout technique (bottom), in infants with and without former BPD (the mode of presentation is the same as that of Figure 1)**.

As PMA increased, a continuous rise (*p *< 0.001) in the end-expiratory lung volume measured either by body plethysmography (FRC_pleth_) or the SF_6 _multiple breath washout technique (FRC_SF6_) was evident in both groups (Figure 7). No statistically significant difference in FRC_pleth _was apparent when former BPD and non-BPD infants were compared, whereas FRC_SF6 _was significantly lower (*p *= 0.036) in the former BPD infants.

In both groups, normalization of FRC_pleth _and FRC_SF6 _values to actual body weight rendered the values of either group near-constant at each of the three measurement time points; no statistically significant between-group difference was evident. Also, when non-BPD and former BPD infants were compared, no statistically significant difference in mean FRC_pleth _values normalized to body weight was evident; the means (with 95% CIs) were 22.7 (21.9-23.4) mL/kg *versus *22.8 (22.0-23.6) mL/kg; *p *= 0.855. This was also true of the normalized mean FRC_SF6 _values: 21.3 (20.3-22.3) mL/kg *versus *20.5 (19.5-21.4) mL/kg; *p *= 0.401.

### Blood gas levels

**Figure 8:**
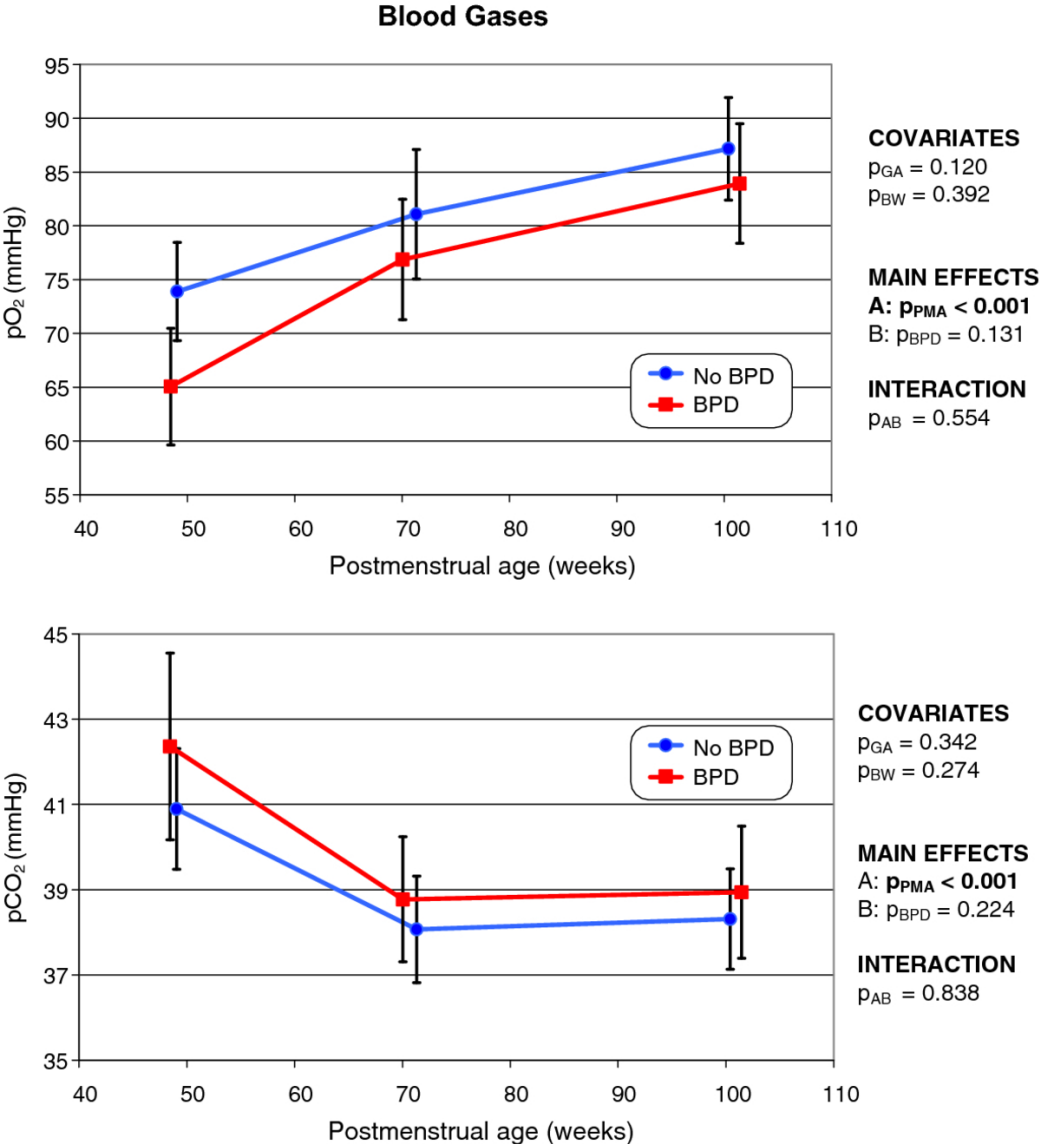
**Changes over time in pO_2 _(top) and pCO_2 _(bottom) levels in infants with and without former BPD (the mode of presentation is the same as that of Figure 1)**.

The development over time of blood gas levels was comparable in either group (Figure 8); no statistically significant influence of gestational age or birth weight was evident. Whereas pO_2 _increased continuously (*p *< 0.001) as PMA rose, pCO_2 _decreased significantly (*p *< 0.001) from the first to the second LFT session and remained near-constant thereafter. Although the mean pO_2 _value of former BPD infants was consistently somewhat lower, and the pCO_2 _somewhat higher, than those of non-BPD infants, the differences did not attain statistical significance. No statistically significant interaction of BPD and PMA was apparent, indicating that differences between infants with and without BPD were constant over time.

## Discussion and Conclusions

In the present study, we have shown that very low birth weight (VLBW) infants (birth weight < 1,500 g) with BPD exhibit reduced somatic growth (in terms of body weight and length) and impairment of some lung function parameters, compared to VLBW infants without BPD, when assessed at the same PMA. With the exception of the tidal breathing parameters V_T _and V'_E_, we found no evidence for catch-up during the first 15 months of life. This is in agreement with the data obtained from sequential lung function measurements performed at 6, 12, and 24 months after birth on 44 infants with moderate-to-severe BPD, which showed that lung function abnormalities persisted [29]. A study by Baraldi et al. [13] of 24 VLBW infants with BPD showed that pulmonary mechanics improved during the first years of life but substantial airway functional impairment remained, as revealed by a low V'max_FRC _The extent to which lung function in BPD survivors improves with age remains controversial [24]. Blayney et al. [30] investigated 32 former BPD infants at mean ages of 7 and 10 years. Those with normal lung function at age 7 years demonstrated normal lung growth whereas those with evidence of impaired lung function at 7 years of age exhibited continued lung growth or repair, or both, during later school years. In another study, Koumbourlis et al. [31] performed repeated lung functional testing of 17 former BPD subjects between the ages of 8 and 15 years and found that although airflow obstruction may persist, this does not deteriorate later in life; an improvement in air trapping over time was evident. A more recent study by Doyle et al. [24] on 147 former VLBW infants, of whom 33 formerly had BPD, showed that, at a mean age of 19 years, those with former BPD had poorer lung function than those without former BPD.

In the present study, the most significant lung function differences between the two patient groups were in the tidal breathing parameters (V_T_, V'_E_), lung compliance and end-expiratory lung volume. Whereas FRC_pleth _did not differ between former BPD and non-BPD infants, FRC_SF6 _(which measures the lung volume in actual communication with the airways) was significantly lower in former BPD compared to non-BPD infants. However, all differences in tidal breathing, lung compliance and FRC values disappeared after normalization of such parameters to actual body weight. This is attributable to the significant somatic growth retardation evident in former BPD infants. Normalization in terms of weight is usually performed after LFT of infants to reduce the extent of (the otherwise high) inter-subject variability. However, in infants experiencing growth retardation, such normalization may lead to overestimation of lung function. Hence, the results should be interpreted with caution. The reasons for the poor growth of preterm infants with former BPD remain unknown, but likely include dysfunction of various organ systems, decreased nutrient intake, and increased energy requirements [31].

Immaturity and BPD independently impair postnatal lung function. Hoo et al. [32] were the first to show that the V'max_FRC _was reduced in preterm infants in the absence of any neonatal disease or therapy. The reduction was attributable to the arrest of lung and airway development. A preliminary study by Gappa et al. [33], measuring V'max_FRC _in premature infants with and without BPD, suggested that prematurity *per se *may be a more important contributor to the observed impairment in lung function than is BPD. This may explain why we did not find any statistically significant difference in V'max_FRC _values between preterm infants with and without former BPD.

Although the measurement of V'max_FRC _is currently the most frequently used lung function test in infants who are unable to cooperate, the effect of the BPD on lung function can not only reduced on the assessment of the small airways. Hjalmarson and Sandberg [10] found in preterm infants with severe BPD a reduced FRC and increased inhomogeneity indices indicating an impaired alveolar gas mixing. Furthermore they found a reduced lung compliance and changes in the breathing pattern; the tidal volume was decreased and the respiratory rate increased. The differences between healthy preterm infants and those with mild-to-moderate BPD were distinctly lower.

Several studies [12,17,34-36] using tracer gas techniques have shown that the FRC is reduced in former BPD infants, in good agreement with the findings of the present study. This reduction in FRC_SF6 _may not necessarily reflect a defect in lung development (the FRC_pleth _values were identical in either group) but rather a reduction in the proportion of the lung volume that participates in pulmonary gas exchange, in turn attributable to structural changes in the lung caused by BPD and the use of mechanical ventilation. All of our BPD infants required invasive mechanical ventilation.

Consistent with the findings of the present study, Hjalmarson and Sandberg [10] found no significant difference in respiratory resistance between former BPD and non-BPD infants. Further, the variation in lung compliance did not persist after normalization to actual body weight. In contrast to studies from the presurfactant era, which predominantly investigated infants born at term [37,38], no difference in the elastic performance of the respiratory system was evident between former BPD and non-BPD preterm infants. This may be explained by the fact that respiratory compliance after birth, in both groups, was very low, as a result of immaturity (as reflected by the low birth weights). Rapid catch-up growth during the first 15 months of life was evident; normal values of approximately 14 mL/kPa/kg [39] were eventually attained. Former BPD infants attained such values somewhat more slowly than did non-BPD infants (Figure 4, top). Baraldi et al. [13] also found that the respiratory compliance of VLBW infants with former BPD became normal at 2 years of age.

Catch-up growth in terms of lung compliance during the first year of life was reported in a study of BPD infants performed by Gerhardt et al. [40] in the presurfactant era 25 years ago).

The cited authors speculated that, in former BPD infants, the increase in compliance evident upon aging was associated with lung growth, and more specifically to the formation of new alveoli. Specific compliance increased with age, as was also the case in our present work (Figure 5, bottom), supporting the interpretation of Gerhardt et al. [40].

As the lung grew, maturation of the breathing pattern was observed in both groups, as shown by an increase in tidal volume and a fall in the respiratory rate. However, a distinct delay in maturation was apparent in former BPD infants. Only a few studies have measured tidal breathing parameters in preterm BPD infants. Previously [41,42], we found that BPD infants showed an increased respiratory rate and elevated minute ventilation, but no change in tidal volume, compared with healthy controls delivered at term. Similar results were obtained by Latzin et al. [12], who compared preterm former BPD infants with preterm healthy newborns at 44 weeks PMA. Hjalmarson and Sandberg [10] described a similar pattern in preterm infants with mild-to-moderate former BPD. Tidal volume (normalized to body weight) was significantly reduced in infants with severe BPD, compared to healthy preterm controls. Although the data did not attain statistical significance, we also noted increases in both respiratory rate and minute ventilation, normalized to body weight at term, in the present study. This breathing pattern is characteristic of stiff lungs and is energetically optimal [43]. However, with increasing age, a decrease in minute ventilation (normalized to body weight) was evident in former BPD infants. This was caused by a decrease in tidal volume. In non-BPD infants, minute ventilation per kilogram of body weight was near-constant. It remains unclear whether the decrease in minute ventilation in former BPD infants is attributable to impairment of respiratory mechanics resulting in a lower FRC, to the existence of a lower respiratory drive, or to a defect in respiration control.

The present study had several strengths and limitations. The strengths are the depth at which LFT was performed; we measured tidal breathing, respiratory mechanical parameters, lung volume, maximal respiratory flow, and blood gas levels. Also, all patients were examined by the same investigator, using the same equipment and protocol. Apart from the known intersubject variability of several of the parameters that we measured, LFT remains highly device- and protocol-dependent. This poses major problems in multicenter studies [17]. The limitations of our study are the retrospective nature of our analysis, the small sample sizes of both groups (limiting our power to detect differences between former BPD and non-BPD infants with statistical significance), and the lack of a control group of healthy infants [44]. It is virtually impossible to recruit such a control group, for both practical and ethical reasons. Because our study was observational in nature, no causal relationships can be deduced from our findings.

In conclusion, the extent of somatic growth, and the evolution of some lung function parameters, of very preterm infants with former BPD lag behind those characteristic of preterm infants without BPD for the first 15 months of life. The differences between the groups in most lung function parameters disappear after the somatic growth retardation of former BPD infants is taken into account. Longitudinal LFT of preterm infants after discharge from hospital may help to identify those at risk of incomplete recovery of respiratory function, which can lead to development of respiratory problems in childhood and adolescence.

## Abbreviations

BPD: Bronchopulmonary dysplasia; C_rs_: Respiratory compliance; FRC_pleth_: Functional residual capacity measured by body plethysmography; FRC_SF6_: Functional residual capacity measured by SF_6 _multiple breath washout; MANOVA: Multivariate analysis of variance; MBW: Multiple-breath inert gas washout; PMA: Postmenstrual age; R_aw_: Airway resistance; RR: Respiratory rate; R_rs_: Respiratory resistance; SF_6_: Sulfur hexafluoride; TB: Tidal breathing; V'_E_: Minute ventilation; V'max _FRC_: Maximal expiratory flow at the functional residual capacity; VLBW: Very low birth weight (< 1,500 g); V_T_: Tidal volume

## Competing interests

The authors declare that they have no competing interests.

## Authors' contributions

GS and CB were primarily responsibility for the study design, data analysis, and writing of the manuscript. SW carried out all measurements of lung function and GS performed the statistical analysis. CCR and HP developed the protocol for lung function testing and had primary responsibility for patient recruitment. All authors read and approved of the final manuscript.

## Pre-publication history

The pre-publication history for this paper can be accessed here:

http://www.biomedcentral.com/1471-2431/12/37/prepub
